# Supplementary material for: Delineating Geographical Regions with Networks of Human Interactions in an Extensive Set of Countries
Source: PLoS One. 2013 Dec 18;8(12):e81707. doi: 10.1371/journal.pone.0081707 (PMC3867326; doi:10.1371/journal.pone.0081707)
Supplement: File S1 — Contains: Table S1. Partition differences to the administrative regions, for alternative algorithms. Figure S1. Cluster overlap index comparing the noiseless partitions with partitions having different levels of noise, for Belgium and Portugal. Figure S2. Partitioning of Portugal with different levels of noise. Figure S3. Partitioning of Portugal with different algorithms. (PDF) [file pone.0081707.s001.pdf]

# SUPPLEMENTARY INFORMATION

## Delineating geographical regions with networks of human interactions in an extensive set of countries

Stanislav Sobolevsky[1,*], Michael Szell[1], Riccardo Campari[1], Thomas Couronné[2], Zbigniew Smoreda[2], Carlo Ratti[1]
**1 Senseable City Laboratory, Massachusetts Institute of Technology, 77 Massachusetts Avenue, Cambridge, MA 02139, USA**
**2 Sociology and Economics of Networks and Services Department, Orange Labs, F-92794 Issy-les-Moulineaux, France**
**∗ E-mail: stanly@mit.edu**

## Definition of partition overlap measures

Consider a set of locations $\mathcal{T}$ of cardinality $n$ and two partitions $\mathcal{C}$ and $\mathcal{C}'$ of $\mathcal{T}$, then the set of all unordered pairs of elements of $\mathcal{T}$ is the union of the sets [1, 2]:

$t_{11}$ is the set of pairs the same community under $\mathcal{C}$ and $\mathcal{C}'$;

$t_{01}$ is the set of pairs not in the same community under $\mathcal{C}$ but under the same community in $\mathcal{C}'$;

$t_{10}$ is the set of pairs in the same community under $\mathcal{C}$ but not under the same community in $\mathcal{C}'$;

$t_{00}$ is the set of pairs not in the same community under $\mathcal{C}$ and $\mathcal{C}'$;

and $n_{11}$, $n_{01}$, $n_{10}$, $n_{00}$ are their respective cardinalities, and $n_{11} + n_{01} + n_{10} + n_{00} = n(n-1)/2$. The $\mathcal{R}$ [3] and $\mathcal{F}$ [4] indices are then given by:

$$\mathcal{R} = \frac{2(n_{11} + n_{00})}{n(n-1)} \qquad \mathcal{F} = \frac{n_{11}}{\sqrt{(n_{11} + n_{10})(n_{11} + n_{01})}} \tag{1}$$

which are two ways of quantifying how well the partitions match pairs of locations. A perfect match between two partitions will have $\mathcal{R}, \mathcal{F} = 1$. For the case of two completely unrelated clusterings, both indices are in general strictly larger than zero, more so for $\mathcal{R}$ [4]. Therefore, to have a baseline, we calculated the average indices over 1000 random reshufflings of locations in given partitionings of administrative regions, denoted by $\bar{\mathcal{R}}_{\mathrm{r}}$ and $\bar{\mathcal{F}}_{\mathrm{r}}$.

The classical measures $\mathcal{R}, \mathcal{F}$ are asymptotically invariant of the problem size $n$, and only weakly $n$-invariant for finite $n$, therefore being well suited for different data sets as in our case. However, their base lines can vary heavily ($\mathcal{R}$ between 0.5 and 0.95, $\mathcal{F}$ between 0 and 0.6), making their linearity and usefulness doubtful even after normalization [1]. To have a measure grounded in another, information-theoretical approach, we also use the variation of information $VI$, defined as

$$VI(\mathcal{C}, \mathcal{C}') = H(\mathcal{C}) + H(\mathcal{C}') - 2I(\mathcal{C}, \mathcal{C}'), \tag{2}$$

where $H(\mathcal{C})$ is the Shannon entropy of partition $\mathcal{C}$ and $I(\mathcal{C}, \mathcal{C}')$ the mutual information between the two partitions $\mathcal{C}$ and $\mathcal{C}'$,

$$H(\mathcal{C}) = -\sum_{i=1}^{k} P(i) \log_2 P(i) \qquad I(\mathcal{C}, \mathcal{C}') = \sum_{i=1}^{k} \sum_{j=1}^{l} P(i,j) \log_2 \frac{P(i,j)}{P(i)P(j)}, \tag{3}$$

where $P(i) = \frac{|C_i|}{n}$ is the probability that an element of $\mathcal{T}$ chosen at random belongs to community $C_i \in \mathcal{C}$, and $P(i,j) = \frac{|C_i \bigcap C'_j|}{n}$ the probability that an element belongs to $C_i \in \mathcal{C}$ and to $C'_j \in \mathcal{C}'$. The

$VI$ has mathematical properties that are in line with our general intuition of what "more different" and "less different" should mean for two clusterings of a set. Although the $VI$ is bounded by $\log_2 n$, we are not making use of its normalized form (division by $\log_2 n$) since it does not allow comparisons between distances obtained on different data sets [1].

# The Algorithm

To the extracted communication networks we apply a standard modularity optimization approach for community detection [5, 6]. The approach scores all the edges of the network according to their relative strength with respect to the weight of the nodes they connect and aims to maximize the cumulative score inside the communities, preferring edges with a positive score and avoiding those with a negative score. The particular optimization algorithm [7] (further referred as "Combo") is a novel enhancement of the technique used by [8]. The idea is an iterative improvement of the partitioning in terms of the modularity score, starting from a trivial case where all nodes are gathered into one community following three steps of improvement: 1) dividing a community into two new communities, 2) joining two communities into one, and 3) shifting a part of one community to another existing community. The technique of splitting the community for the purpose of the first and third operations is based on the Kernigan-Lin approach [9] in a way similar to the refinement procedure suggested by [5]. The algorithm effectively avoids getting stuck in local maxima and usually produces the best modularity scores compared to the majority of modularity optimization algorithms known so far including Newman's greedy optimization heuristic [10], Newman's spectral optimization with refinement [5], simulated annealing [11] and another fast aggregation algorithm for large networks recently suggested by [12] known as Louvain method. It allows to handle networks up to 5,000-10,000 nodes in a reasonable time which corresponds to the dimension of our networks.

We use this particular algorithm here for an improved partitioning quality for the large networks, see Fig. S3 and Table S1. Although major qualitative properties of the partitioning remain the same and do not considerably depend on the particular algorithm used, see also section "Independence on the algorithm" below, as high as possible modularity scores are reached, together with an improved quality of boundary definitions and lowered levels of noise (less "islands").

The intriguing property of the modularity optimization approach is that the resulting network division has no predetermined number of partitions. Only the raw topological information of the input network determines the range of communities detected – the algorithm may detect any number of communities between 1 (the full network) and $N$, the number of nodes (where each community is made up of one single node). Further, the algorithm does not fix the sizes nor the distribution of sizes of the detected groups, and it is not limited by any spatial constraints. Note that on the one hand, in cases where the algorithm produces boundaries which match official boundaries this can carefully be interpreted as having a "natural" validation. On the other hand if the algorithm does *not* so, the reasons – apart from a genuine deviation of human interaction regions from official boundaries – could also include low population density near the border making boundaries visually floating but leaving modularity scores practically unchanged, and other possible minor statistical fluctuations. Due to such influences, the boundaries produced by the algorithm cannot always be treated as definitely exact, as they may be shifted slightly. However, the cores of detected regions have been shown to be stable [8].

# Stability analysis

The findings presented in the main text are based on a particular partitioning algorithm, and on a number of data sources possibly featuring different measurement errors or modes of collection. Therefore, here we proceed with an analysis of the variations of outcomes from running the algorithm multiple times, and of the strength of the partitions under perturbations, to understand the robustness and limits of the

approach.

## Robustness of algorithm

The used “Combo” method involves a stochastic element and therefore does not necessarily produce the same partitioning result with every run [7]. To test possible variations, we ran the algorithm 10 times for Portugal. All 10 results gave exactly the same results, showing that for the case of large spatial networks of communications there are practically no variations.

## Independence on the algorithm

To test the robustness of the partitions in respect to the clustering algorithms used, we applied three additional standard algorithms besides our “Combo” method: the Louvain method [12], Newman’s greedy optimization heuristic [10], Newman’s spectral algorithm with refinement [5]. Resulting clustering overlap indices with the administrative regions are shown in Table S1. Generally the indices show comparable levels of overlap, in most cases slightly increasing for partitioning with better modularity; while the index *VI* is lower when the similarity is higher. The finding of similarity to official regions is also qualitatively independent of the algorithm used, as well as the “natural balance” of the number of regions, see number of communities in Table S1. Finally, see Fig. S3, using the examplary case of Portugal (but holding for the other countries as well), the geographical cohesiveness of resulting communities is also confirmed by all algorithms − apart from some minor noise, resulting communities consist of spatially adjacent locations. Therefore, the main qualitative properties of the partitioning seem to be independent of the algorithm, but using higher performance algorithms helps improving the overall quality of the delineation of boundaries and reducing noise levels.

## Robustness of network data

To probe the robustness of the resulting partitions to noise in the data collection, we ran community detection on several realizations of each network, each of which we perturbed with increasing levels of noise, and compared the results with official administrative areas.

We devised the perturbations based on a simple model for the formation of the network: we assumed that, for each of the $N$ phone calls, source and destination nodes are chosen according to a multinomial distribution, in which connectivity likelihoods $f_{ij}$ are approximated by the observed fractions $\hat{f}_{ij} = N_{ij}/N$ for boostrapping purposes.

As a simplifying step, we decided to treat each pair of nodes as independent of all other pairs, and thus governed by a binomial, which is the corresponding marginal distribution.

As a further step to ensure the numerical feasibility of the generation of each network, we approximated the binomial distribution by using the corresponding Gaussian, in accordance with the de Moivre-Laplace theorem.

Perturbed networks were thus generated as

$$N'_{ij} = N_{ij} + wn\sqrt{N_{ij}},$$

where $n$ is a normal random variable with zero average and unit standard deviation, and $w$ is an arbitrary weight which we varied to obtain a better understanding of the solidity of partitioning.

When considering duration of calls $D_{ij}$, the generated network incurs an additional factor:

$$D'_{ij} = D_{ij} + wn\sqrt{\langle D\rangle D_{ij}},$$

where $\langle D\rangle = \sum_{ij} D_{ij}/N$ is the average duration of each call.

For each network, we varied $w$ from 0 (original network) to 10.5; then, for each $w$ we generated 10 networks, computed the corresponding community structure, and compared it to the original administrative area using the cluster overlap index $\mathcal{R}$.

Results are shown in Supplementary Fig.S1. It should be noted that employing the alternative indices, $\mathcal{F}$ and $VI$, yields precisely the same qualitative behaviour.

In the case of Belgium, moderate perturbations ($w \simeq 2$) of the network result in sizable variations in the resulting partition, which stabilize when the noise increases. Even higher levels precipitate the overlap, as the underlying network is overcome by fluctuations. Similar analyses apply to Portugal and other networks.

The values of $w$ at which steep slopes happen in Supplementary Fig.S1 can be interpreted as levels of noise which are just strong enough to shake subsets of links responsible for the stability of the partition; this point of view also justifies the corresponding net increase of standard deviation for $\mathcal{R}$. However in all cases values of $w$ for which the variations become considerable are substantially higher than the normal average value 1 expected for the random model introduced above.

The variations we observe in the resulting partitioning need not be strictly interpreted on the base of the multinomial model: while the latter describes noise from one particular process, variations in link weights can also do away with systematic biases from other sources, such as geographical variations in market share, or conspicuous deviations of $\hat{f}_{ij}$, the observed fractions, from the "real" likelihood $f_{ij}$. It is in this spirit that we can understand increases in $\mathcal{R}$ as the result of a more reliable network.

# References


1. Meila M (2007) Comparing clusterings–an information based distance. Journal of Multivariate Analysis 98: 873 - 895.

2. Brandes U, Delling D, Gaertler M, Görke R, Hoefer M, et al. (2007) On finding graph clusterings with maximum modularity. In: Graph-Theoretic Concepts in Computer Science. Springer, pp. 121–132.

3. Rand W (1971) Objective criteria for the evaluation of clustering methods. Journal of the American Statistical Association 66: pp. 846-850.

4. Fowlkes E, Mallows C (1983) A method for comparing two hierarchical algorithms, volume 78. Journal of the American Statistical Association, 553-569 pp.

5. Newman M (2006) Modularity and community structure in networks. Proceedings of the National Academy of Sciences 103: 8577–8582.

6. Fortunato S (2010) Community detection in graphs. Physics Report 486: 75–174.

7. Sobolevsky S, Campari R, Belyi A, Ratti C (2013) A general optimization technique for high quality community detection in complex networks. arXiv:13083508 .

8. Ratti C, Sobolevsky S, Calabrese F, Andris C, Reades J, et al. (2010) Redrawing the map of Great Britain from a network of human interactions. PLoS One 5: e14248.

9. Kernigan B, Lin S (1970) An efficient heuristic procedure for partitioning graphs. Bell Syst, Tech J 49: 291–307.

10. Newman M, Girvan M (2004) Finding and evaluating community structure in networks. Physical Review E 69.

11. Guimerà R, Sales-Pardo M, Amaral L (2004) Modularity from fluctuations in random graphs and complex networks. Phys, Rev E70(2): 025101.

12. Blondel V, Guillaume J, Lambiotte R, Lefebvre E (2008) Fast unfolding of communities in large networks. J Stat Mech 10008.

**Table S1. Partition differences to the administrative regions, for alternative algorithms.** Clustering indices and numbers of partitions are consistent, i.e. qualitatively independent of the algorithm, but Combo provides the highest modularity scores. Due to reasons of data access we were not able to execute the alternative algorithms for the France data set.

| $\mathcal{R}$ | $\bar{\mathcal{R}}_\mathrm{r}$ | Combo | Louvain | Greedy | Spectral |
|---|---|---|---|---|---|
| France | 0.860 | 0.985 | – | – | – |
| UK | 0.809 | 0.955 | 0.904 | 0.948 | 0.947 |
| Italy | 0.883 | 0.957 | 0.954 | 0.957 | 0.961 |
| Belgium | 0.819 | 0.932 | 0.935 | 0.931 | 0.931 |
| Portugal | 0.677 | 0.885 | 0.865 | 0.885 | 0.889 |
| Ivory Coast | 0.739 | 0.870 | 0.853 | 0.885 | 0.881 |
| Saudi Arabia | 0.794 | 0.904 | 0.893 | 0.899 | 0.899 |
| $\mathcal{F}$ | $\bar{\mathcal{F}}_\mathrm{r}$ | | | | |
| France | 0.076 | 0.900 | – | – | – |
| UK | 0.107 | 0.772 | 0.558 | 0.741 | 0.731 |
| Italy | 0.063 | 0.647 | 0.624 | 0.633 | 0.675 |
| Belgium | 0.101 | 0.647 | 0.655 | 0.640 | 0.621 |
| Portugal | 0.203 | 0.697 | 0.657 | 0.712 | 0.695 |
| Ivory Coast | 0.154 | 0.505 | 0.460 | 0.592 | 0.573 |
| Saudi Arabia | 0.117 | 0.606 | 0.560 | 0.591 | 0.544 |
| *VI* | $\log_2 n$ | | | | |
| France | 14.12 | 0.676 | – | – | – |
| UK | 12.21 | 1.322 | 2.432 | 1.477 | 1.493 |
| Italy | 7.79 | 1.349 | 1.408 | 1.464 | 1.233 |
| Belgium | 9.20 | 1.538 | 1.549 | 1.608 | 1.720 |
| Portugal | 11.08 | 1.465 | 1.819 | 1.572 | 1.468 |
| Ivory Coast | 10.19 | 2.054 | 2.326 | 1.952 | 2.113 |
| Saudi Arabia | 8.98 | 2.036 | 2.262 | 2.264 | 2.561 |
| Num. regions | | | | | |
| France | 22 | 21 | – | – | – |
| UK | 11 | 16 | 13 | 14 | 15 |
| Italy | 21 | 21 | 21 | 23 | 21 |
| Belgium | 11 | 12 | 13 | 12 | 14 |
| Portugal | 5 | 7 | 7 | 6 | 8 |
| Ivory Coast | 19 | 11 | 11 | 10 | 10 |
| Saudi Arabia | 13 | 12 | 12 | 12 | 16 |
| Modularity | | | | | |
| UK | – | 0.6206 | 0.5764 | 0.6140 | 0.6125 |
| Italy | – | 0.7224 | 0.7218 | 0.7222 | 0.7215 |
| Belgium | – | 0.7423 | 0.7421 | 0.7417 | 0.7388 |
| Portugal | – | 0.4904 | 0.4451 | 0.4702 | 0.4874 |
| Ivory Coast | – | 0.3743 | 0.3638 | 0.3699 | 0.3702 |
| Saudi Arabia | – | 0.4803 | 0.4734 | 0.4797 | 0.4778 |

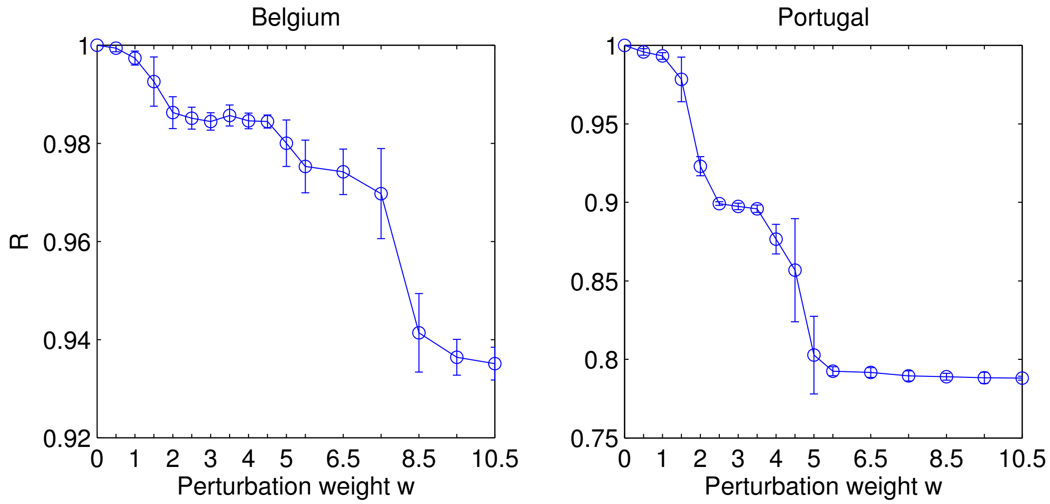


**Figure S1. Cluster overlap index $\mathcal{R}$ comparing the noiseless partitions with partitions having different levels of noise, for Belgium and Portugal.** For each noise level $w$ we produced 10 realizations of the perturbed network. Markers and error bars denote the means and standard deviations of these realizations, respectively. Up to a noise level of 1, there is practically no difference in the produced partitions. For higher noise levels, $\mathcal{R}$ drops lower in Portugal than in Belgium because of the difference in spatial resolutions $n$.

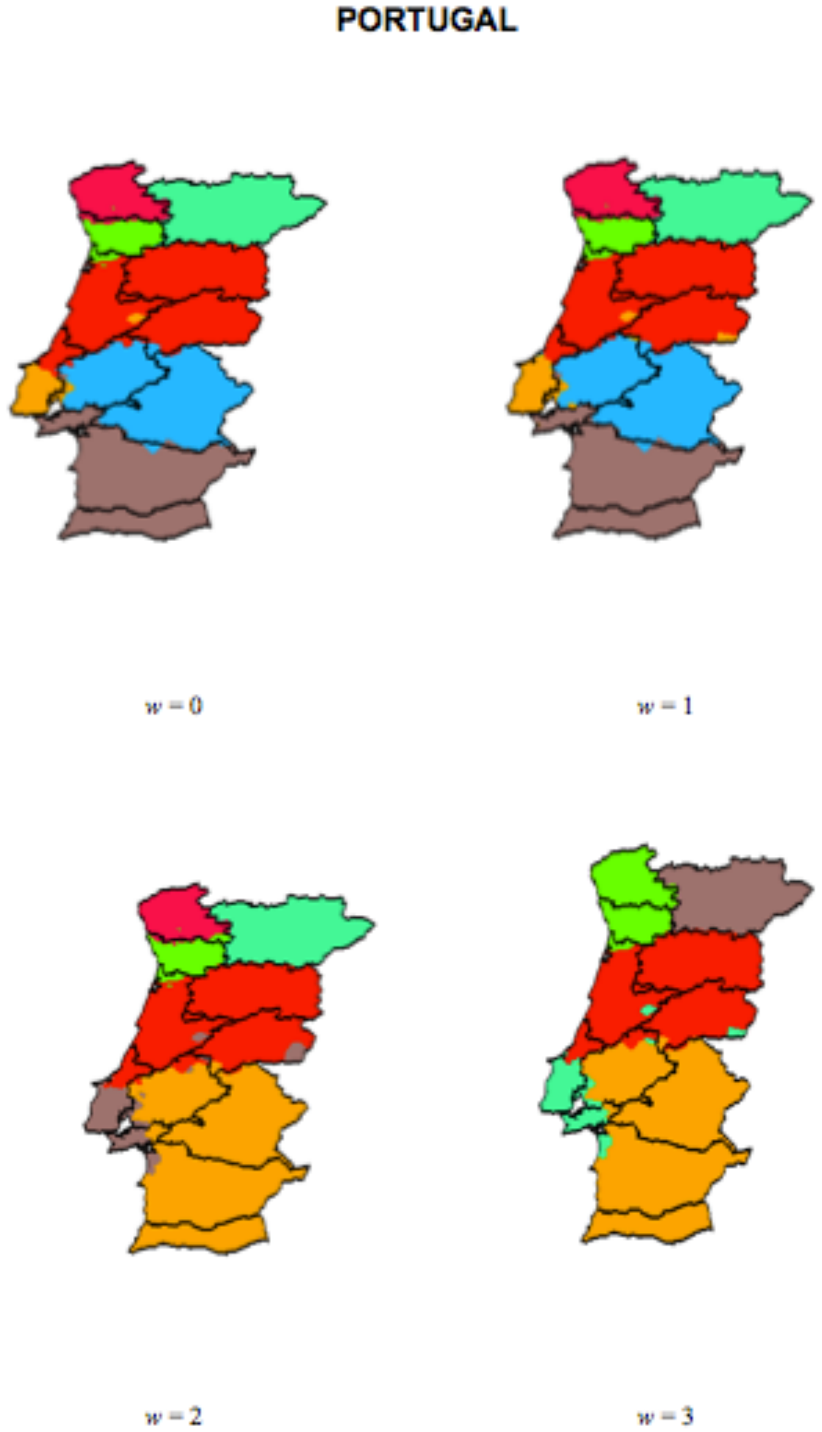


**Figure S2. Partitioning of Portugal with different levels of noise.** With increasing noise, pairs of partitions start to merge together. One can also notice the appearance of small disjoint "islands".

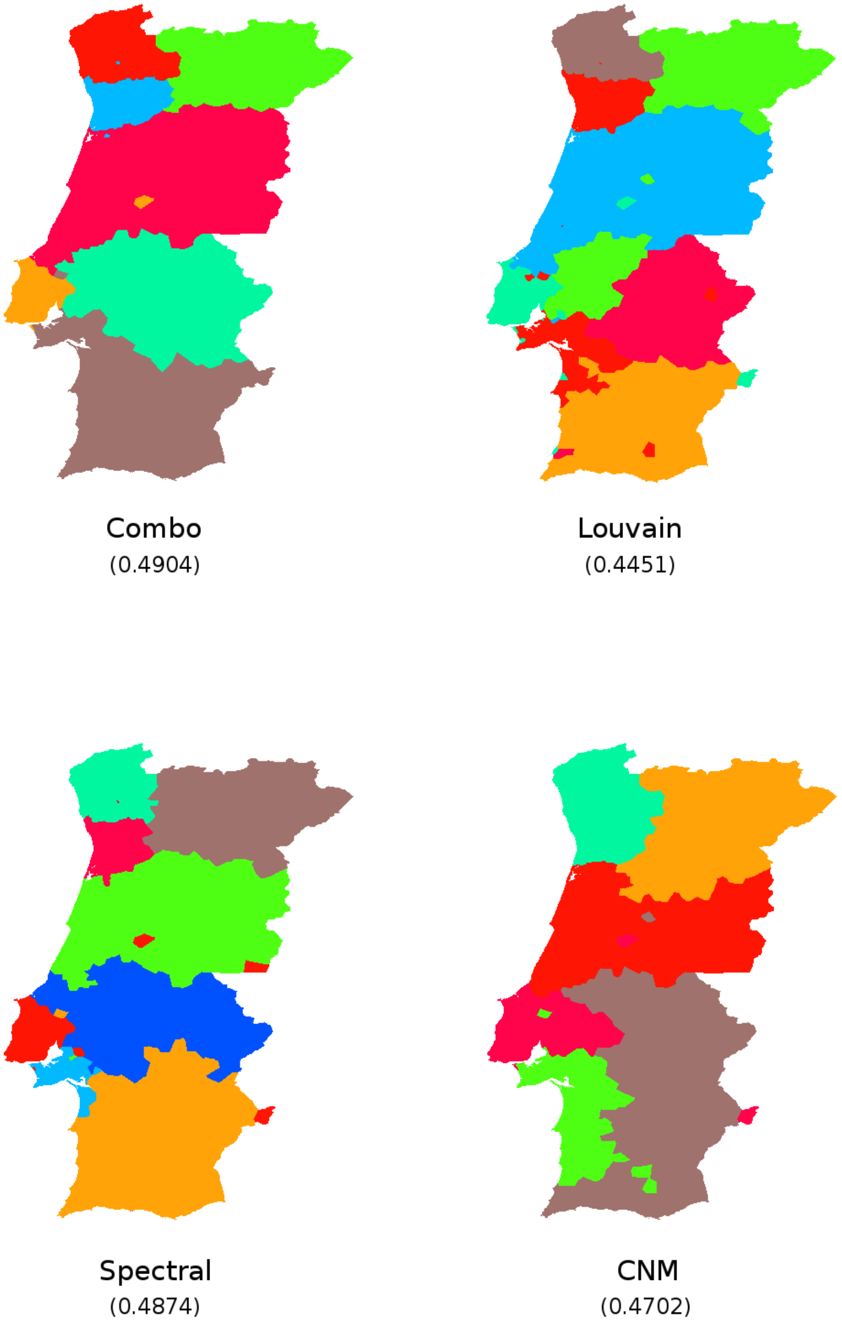


**Figure S3. Partitioning of Portugal with different algorithms.**
